# Advanced biomaterial agent from chitosan/poloxamer 407-based thermosensitive hydrogen containing biosynthesized silver nanoparticles using *Eucalyptus camaldulensis* leaf extract

**DOI:** 10.1371/journal.pone.0291505

**Published:** 2023-10-20

**Authors:** Suttiwan Wunnoo, Ana C. Lorenzo-Leal, Supayang P. Voravuthikunchai, Horacio Bach

**Affiliations:** 1 Faculty of Science, Division of Biological Science, Prince of Songkhla University, Hat Yai, Songkhla, Thailand; 2 Center of Antimicrobial Biomaterial Innovation-Southeast Asia, Prince of Songkhla University, Hat Yai, Songkhla, Thailand; 3 Division of Infectious Disease, Department of Medicine, University of British Columbia, Vancouver, BC, Canada; Brandeis University, UNITED STATES

## Abstract

**Context:**

The emergence of multidrug-resistant (MDR) pathogens poses a significant challenge for global public health systems, increasing hospital morbidity and mortality and prolonged hospitalization.

**Objective:**

We evaluated the antimicrobial activity of a thermosensitive hydrogel containing bio-synthesized silver nanoparticles (bio-AgNPs) based on chitosan/poloxamer 407 using a leaf extract of *Eucalyptus calmadulensis*.

**Results:**

The thermosensitive hydrogel was prepared by a cold method after mixing the ingredients and left at 4°C overnight to ensure the complete solubilization of poloxamer 407. The stability of the hydrogel formulation was evaluated at room temperature for 3 months, and the absorption peak (420 nm) of the NPs remained unchanged. The hydrogel formulation demonstrated rapid gelation under physiological conditions, excellent water retention (85%), and broad-spectrum antimicrobial activity against MDR clinical isolates and ATCC strains. In this regard, minimum inhibitory concentration and minimum microbial concentration values of the bio-AgNPs ranged from 2–8 μg/mL to 8−128 μg/mL, respectively. Formulation at concentrations <64 μg/mL showed no cytotoxic effect on human-derived macrophages (THP-1 cells) with no induction of inflammation.

**Conclusions:**

The formulated hydrogel could be used in biomedical applications as it possesses a broad antimicrobial spectrum and anti-inflammatory properties without toxic effects on human cells.

## Introduction

Rising antibiotic-resistant infections are a significant challenge for global public health systems. Multidrug-resistant (MDR) microorganism infections have become a global emergency with profound public health concerns. Most infections caused by MDR pathogens can result in treatment failure and death. Although MDR bacteria are primarily associated with nosocomial infections, some are now causing community-acquired infections, increasing the population at risk [1]. A recent study revealed that 1.27 million deaths worldwide in 2019 were directly related to antibiotic resistance and MDR. The most severe antibiotic-resistant pathogens are ESKAPE or *E**nterococcus faecium*, *S**taphylococcus aureus*, *K**lebsiella pneumoniae*, *A**cinetobacter baumannii*, *P**seudomonas aeruginosa*, and *Enterobacter* spp. [2, 3]. The antibiotic resistance of clinical isolates of *Candida* strains is also rising, including MDR strains [4, 5].

Microorganism infections are commonly affected by microbial colonization with the potential of biofilm development on wound dressings and indwelling medical devices such as endotracheal tubes, catheters, and vascular lines [6, 7]. Biofilms protect bacterial cells from antibiotics and other antimicrobial agents and facilitate the bacterial transformation and conjugation process more efficiently, promoting the transmission of resistance genes [8].

The synthesis of biofilms by microorganisms constitutes a challenge for treatment as the antibiotic administration has to be increased for a favorable outcome [9, 10]. To address these concerns, silver nanoparticles (AgNPs) can effectively prevent MDR pathogens because it has broad-spectrum antimicrobials with various modes of action, such as microbial membrane rupture and metabolic function disruption, inducing cell death [11]. Furthermore, NPs can penetrate the exopolysaccharide matrix and destroy bacterial cells within biofilms due to their small size ranging between 1–100 nm [12].

Among the approaches used to produce AgNPs, plant-based synthesis is a promising method for synthesizing AgNPs since it is cost-effective, environmentally friendly, biocompatible, and has antibacterial activity against MDR pathogens. For example, bio-AgNPs using *Catharanthus roseus* and *Azadirachta indica* as reducing and capping agents showed good antibacterial activity against MDR microorganisms isolated from wound infections, including enhanced wound-healing [13].

AgNPs embedded into polymeric hydrogels are currently used in various biomedical applications, especially drug delivery. Moreover, temperature-sensitive hydrogels are popular because they can be injected and formed into inaccessible localization areas without additional external stimuli, control active drug release, and possess high mucoadhesive properties [14, 15].

Pluronic^®^ or poloxamers are triblock copolymers with an excellent ability to form gels at physiological temperatures and low toxicity [16]. However, poloxamers have poor mechanical strength, resulting in rapid gel erosion in aqueous media that limits their sustained diffusion qualities [17]. Combining poloxamers with mucoadhesive polymers, such as chitosan, showed a gel erosion modification and enhanced poloxamer adhesive properties [17, 18].

Chitosan is a natural and abundant polymeric material obtained from chitin, a fungi cell wall component, and crustaceans’ exoskeleton. Chitosan has shown high biocompatibility and biodegradability qualities. Amongst its features, chitosan is a matrix-forming material that can reach NP sizes. The chitosan mechanism is based on the interaction of its protonated glucosamine groups with cell membranes [21].

When incorporated into the hydrogels, it can serve as a scaffold biomaterial, including drug delivery [19]. According to a recent study, AgNPs-loaded chitosan-PEG hydrogel demonstrated higher porosity, greater water vapor transition rate, and sustained release of AgNPs, with good antimicrobial activity. This nanocomposite also enhanced wound healing efficiency in rabbits with diabetes compared to the bare chitosan-PEG hydrogel used as a control [20].

Here, our study aimed to develop chitosan/poloxamer 407-based thermosensitive hydrogel loaded with biosynthesized AgNPs using *Eucalyptus camaldulensis* leaf extract (bio-AgNPs CS/P407 hydrogel) for a potential biomedical application. The use of plant extracts in synthesizing AgNP methods is one of the most expanding because of their safe, eco-friendly approach, broad-spectrum antimicrobial activity, and easily scaled‐up technology. In addition, phytochemicals discovered in plant crude extracts facilitate various biological activities of NPs [21, 22].

Our recent study found that the extract of *E*. *camaldulensis* leaf, a by-product from paper industries, contains bioactive components such as polyphenols, carboxylic acids, and proteins known to reduce Ag^+^ to Ag^0^ and stabilize bio-AgNPs [23]. The effect of bio-AgNPs CS/P407 hydrogel was investigated against significant MDR clinical isolates and ATCC strains, including Gram-positive and -negative bacteria and fungi. The hydrogel’s cytotoxicity and immune response effects were evaluated on THP-1 cells, a human monocytic cell line, as well as the impact of the nanocomposite on the inflammatory response of macrophages.

## Materials and methods

### Materials

Silver nitrate (AgNO_3_), chitosan, poloxamer 407, acetic acid, and sodium hydroxide were purchased from Sigma-Aldrich (USA). Mueller-Hinton broth (MHB) for bacterial culturing was from B&D Bioscience (B&D, San Jose, California). Sabouraud dextrose broth (SDB) for yeast culturing was purchased from Gibco, UK. RPMI, fetal calf serum, L-glutamine, penicillin, and streptomycin for culturing human cells were purchased from Invitrogen (Waltham, MA, USA).

### Preparation of *Eucalyptus camaldulensis* leaf extract

*Eucalyptus camaldulensis* Dehnh. (Myrtaceae) leaves were collected from Saraburi Province, Thailand, and identified at the Thai Traditional Medicine faculty, Prince of Songkla University, Thailand. Fresh leaves of *E*. *camaldulensis* were washed and air-dried, and then the dried leaves were ground to powder. The leaf powder (10 g) was soaked in 100 mL of sterile water and boiled at 100°C for 30 min. After cooling, the solution was filtered with Whatman No. 1 filter paper and freeze-dried. The composition of the extracts used in this study has been reported in [24].

### Microorganisms and culture conditions

The resistance profiling of the MDR clinical isolates used in this study has been described in [25]. Briefly, the isolates included *S*. *aureus* (resistance: ciprofloxacin, clindamycin, erythromycin, oxacillin, and penicillin), *K*. *pneumoniae* (resistance: ertapenem and meropenem), *A*. *baumannii* (resistance: meropenem), and *P*. *aeruginosa* (resistance: amikacin, ceftazidime, ceftizoxime, piperacillin-tazobactam, and tobramycin). ATCC reference strains included *S*. *aureus* ATCC 700788, multidrug-resistant *S*. *aureus* (MRSA, ATCC BAA41), *S*. *epidermidis* ATCC 14990, *A*. *baumannii* ATCC 17961, *P*. *aeruginosa* ATCC 33354, *E*. *coli* ATCC 25922 and *Candida albicans* ATCC 14053. Bacterial and yeast strains were sub-cultured overnight at 37°C on MHB and SDB, respectively, supplemented with 15% agar (B&D). A single colony of each pathogen from solidified MHB and SDH was transferred to fresh MHB and SDB and incubated overnight at 37°C for bacterial strains and 30°C for *C*. *albicans*.

### Biosynthesis of silver nanoparticles

The bio-AgNPs were prepared with a method used earlier with some modifications [23]. Briefly, 0.5 mM of silver nitrate (Sigma-Aldrich, US) solution was added into deionized water containing the *Eucalyptus* extract (25 mg/mL), and the mixture solution was heated at 60°C for 30 min. After cooling, the mixture solution was centrifuged at 30,000 rpm for 30 min at 4°C. The pellet of bio‐AgNPs was dried using N_2_, and the quantity of the bio-AgNPs was determined by weight. The NPs stock was prepared at a concentration of 5 mg/mL. After that, bio‐AgNPs were dissolved in deionized water and kept under dark conditions at room temperature for further use. The physical properties of bio‐AgNPs were investigated using UV‐visible spectroscopy in the range of 300–800 nm (Epoch, BioTek plate reader, Winooski, VT, USA). Size distribution, polydispersity index (PDI), and zeta potential were assessed by dynamic light scattering (DLS, Litesizer™ 500 from Anton Paar, Gratz, Austria).

### Chitosan/poloxamer 407-based thermosensitive hydrogel loaded with biosynthesized AgNPs

Chitosan solution (0.5% w/v) was prepared in 0.5% acetic acid solution under stirring for 1 h and kept overnight in a refrigerator. Thermosensitive chitosan/poloxamer 407 hydrogel was produced by a cold method. Briefly, with gentle stirring, poloxamer 407 (24% w/v) was added to the cold chitosan solution. The mixture solution was left overnight at 4°C to ensure the complete solubilization of poloxamer 407. Then, the mixture solution was adjusted to pH 7.0 with 1 M NaOH solution. The chitosan/poloxamer 407-based hydrogel was gently mixed with 10% v/v of the NP solution to achieve the final formulation, 500 mg/mL of bio-AgNPs were finally present in the hydrogel.

In order to study the hydrogel network structures, bio-AgNPs-loaded hydrogel was freeze-dried and sliced. Scanning electron microscopy (SEM) at 10 kV accelerating voltage (Quanta 400 FEG; FEI, USA) was used to analyze the network structure. Energy-dispersive X-ray spectroscopy was used to assess the dispersion coverage of the bio-AgNPs within the hydrogel at a surface area of 100 μm^2^.

### Water content calculation

The water loss rate of hydrogels was used to investigate the water content of bio-AgNPs-loaded CS/P407 hydrogel. The hydrogel formulation of 5 g was poured into a 60 × 15 mm diameter plate without a lid and incubated at 37°C. The plates were weighed at 1, 2, 3, 4, 5, 6, 7, 8, 9, 10, 11, 12, 16, 18, 20, 24, and 48 h to calculate the water loss rate. The percentage of water loss rate was defined as (sample_0_—sample_t_)/5 g × 100, where sample_0_ and sample_t_ are the net weight of the plates with the hydrogel formulation at time 0 and sampled time (t), respectively.

### Antimicrobial activity

The antimicrobial activity of bio-AgNPs-loaded CS/P407 hydrogel was determined by broth dilution method following Clinical and Laboratory Standards Institute guidelines of CLSI [26]. A two-fold dilution of the hydrogel in MHB was prepared in a sterile 96-well plate to obtain final concentrations ranging between 1−256 μg/mL. The microorganism suspensions were adjusted to a 0.5 McFarland standard at OD 600 nm to a final concentration of 2 × 10^6^ CFU/well. The plates were incubated at 37°C for 18 h. The minimum inhibitory concentrations (MIC) values were determined as the lowest concentration of the antimicrobial agent that produced a visible inhibition of bacterial growth. The minimum bactericidal concentration (MBC) values were defined as the lowest concentration that bacterial cells were killed, showing no colony growth on the culture medium.

### Cytotoxicity

THP-1 cells (ATCC TIB-202) were used to determine the cytotoxicity of bio-AgNPs and bio-AgNPs-loaded CS/P407 hydrogel. Cell viability was evaluated by measuring cellular metabolic activity using an MTT assay. The cells were grown in a 75 cm^2^ tissue culture flask containing RPMI supplemented with 10% fetal calf serum (v/v), 1% L-glutamine (v/v), 1% penicillin-streptomycin (v/v), and 0.2% fungizone (v/v) until maintained 1 × 10^5^ to 1 × 10^6^ cells/mL. THP-1 cells were seeded into a 96-well plate at 1 x 10^5^ cells/well density after adding 40 ng/mL phorbol myristate acetate to stimulate monocyte activation and incubated at 37°C in an atmosphere of 5% CO_2_ for 24 h. After incubation, the adhered cells were treated with different concentrations (2−128 μg/mL) of bio-AgNPs and the hydrogel (100 μL) and incubated at 37°C for 24 h with 5% CO_2_. Positive control wells were treated with 10% SDS (Merck). The color background of the formulation was subtracted from the reading. After overnight incubation, the culture media was removed, and MTT (100 μL from a concentration of 5 mg/mL) was added for 4 h. Then, 100 μL of the solubilization solution (20% w/v sodium dodecyl sulfate in 50% dimethylformamide solution containing 2.5% acetic acid and 2.5% 1M HCl) was added. The plates were incubated at 37°C overnight, and the next day, the absorbance was measured at 570 nm on a microplate reader (Epoch, BioTek). The percentage of cell viability was calculated according to the equation: [OD values of sample absorbance/OD values of control absorbance] × 100.

### Immune response assay

THP-1 cells were prepared as described above. After 24 h of incubation, the adhered cells were treated with bio-AgNPs and bio-AgNP CS/P407 hydrogel at concentrations ranging from 8−64 μg/mL. The final volume of each well was 300 μL, and the positive control was 1 μg/mL of LPS from *E*. *coli*. The plates were incubated for 24, 48, and 72 h at 37°C supplemented with 5% CO_2_. The supernatant was transferred to another 96-well plate and placed at −20°C until testing.

The levels of cytokines, interleukin-6 (IL-6), tumor necrosis factor-α (TNF-α), and interleukin 10 (IL-10) were estimated by cytokine ELISA kit from B&D according to the manufacturer’s instructions. After that, a solution of the developer TMB (100 μL) was added to the plates, and the reaction was stopped with 50 μL of 1M H_2_SO_4_. The plates were read at 450 nm using a microplate reader (Epoch, BioTek).

### Statistical analysis

All data are expressed as the mean value of three independent experiments ± the standard deviation (SD). Statistical analysis was carried out using GraphPad Prism Software version 9 by one‐way analysis of variance followed by Dunnett’s test. A significant difference was defined as p < 0.05.

## Results and discussion

### Characterization of bio-AgNPs

**Fig 1:**
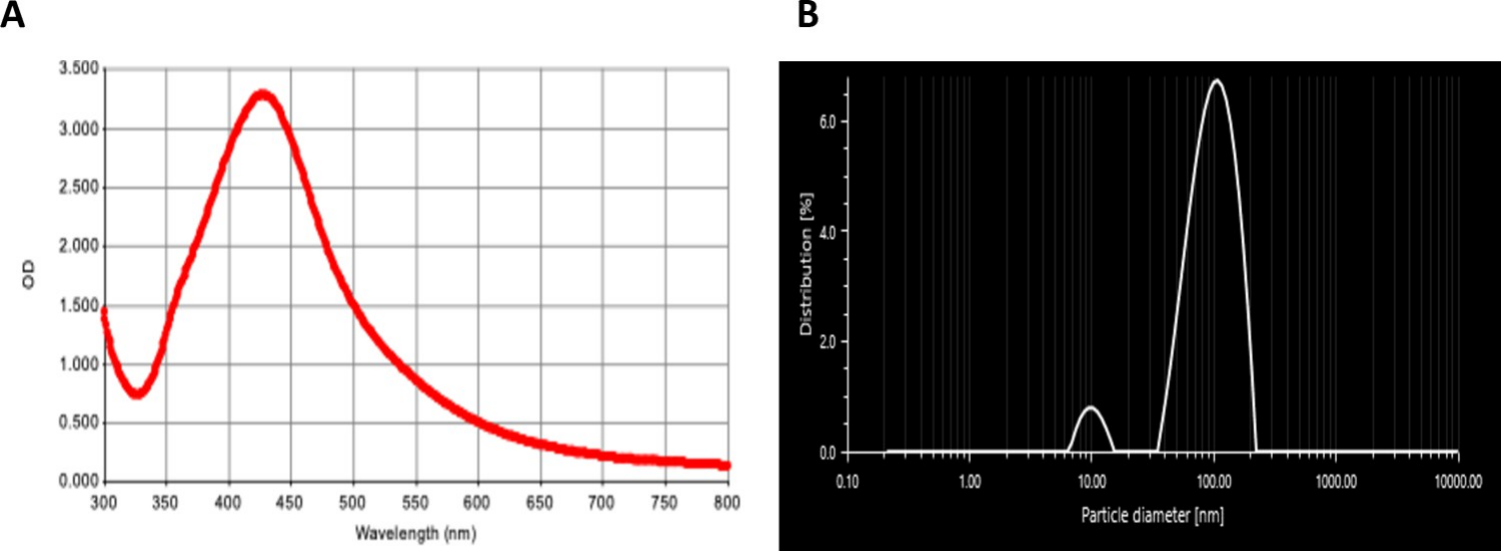
Characterization of bio-AgNPs using aqueous *Eucalyptus camaldulensis* leaf extract by (A) UV–visible spectroscopy and (B) size distribution by dynamic light scattering.

The biosynthesized NPs were prepared in a one-step green method using *E*. *camaldulensis* leaf extract as a reducing and stabilizing agent at 65°C for 30 min. The mixture changes color from light brown to yellowish brown, indicating the formation of AgNPs due to Ag^+^ reduction to Ag^0^. UV-vis spectroscopy with an absorption peak at 420 nm confirmed the production of bio-AgNPs (Fig 1A). DLS analysis showed that the effective diameter of the produced AgNPs was 76.12 nm (Fig 1B). PDI value was used to estimate the stability of colloids and the average uniformity of the particle size [27]. A standard definition of the distribution of size populations within a given sample is the weight average (M_w_) divided by the molecular weight average (M_n_) (PDI = M_w_/M_n_). A sample is considered highly monodisperse when PDI is ≤ 0.1, while the values of 0.1–0.4 and > 0.4 are considered moderately and highly polydisperse, respectively [28].

In this study, bio-AgNPs had a PDI of 0.23, demonstrating that they are moderately dispersed. Bio-AgNPs displayed a zeta potential of -29 mV, indicating that the NPs were stable due to electrostatic repulsions that prevented scattered particle aggregation. Our earlier study using the same methodology for the synthesis of the bio-AgNPs, showed that *E*. *camaldulensis* leaf extract contains the O–H functional group of polyphenols and N–H functional groups of primary and secondary amines of amino acids, peptides, and proteins, as well as the C–O functional group of alcohols, esters, carboxylic acids, and anhydrides [23]. These phytochemicals play a crucial role in reducing the valency of Ag^+^ to Ag^0^ and preventing their agglomeration [29, 30].

### Characterization of bio-AgNPs-loaded CS/P407 hydrogel

**Fig 2:**
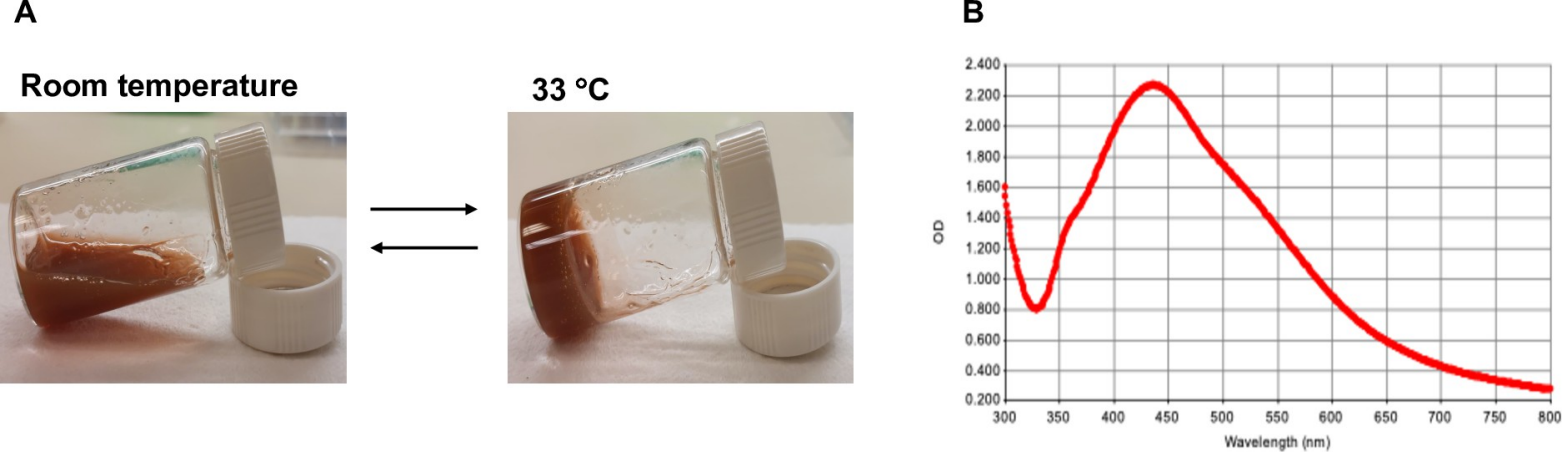
Characterization of chitosan/poloxamer 407-based thermosensitive hydrogel loaded with bio-AgNPs using *E*. *camaldulensis* leaf extract by (A) UV-VIS spectroscopy and (B) phase transition at room temperature (24°C) and gelation temperature (33°C).

**Fig 3:**
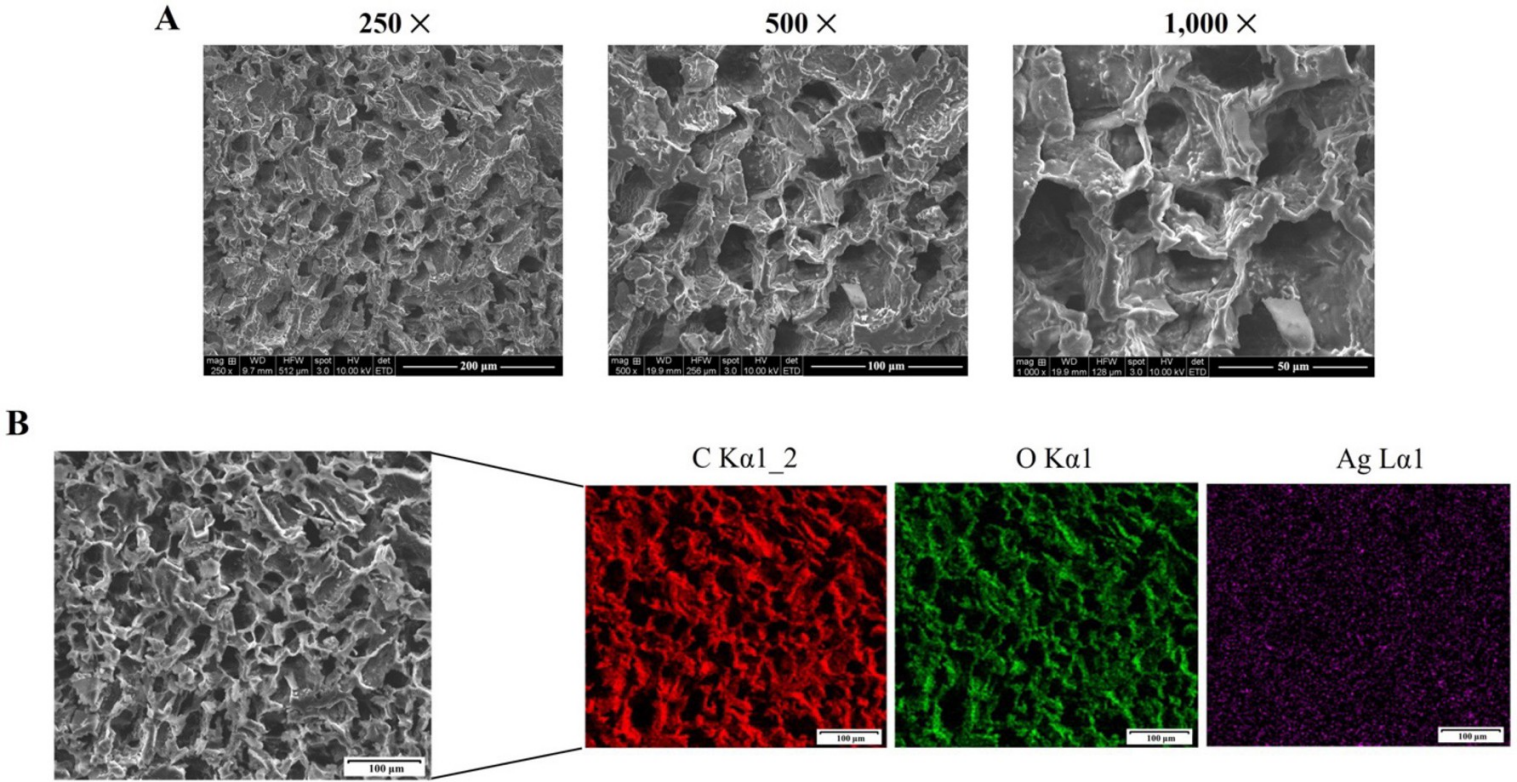
(A) Scanning electron micrographs of chitosan/poloxamer 407-based thermosensitive hydrogen containing biosynthesized silver nanoparticles using aqueous *Eucalyptus camaldulensis* leaf extract (bio-AgNPs CS/P407 hydrogel) at × 250, × 500, and × 1000 magnification, scale bars = 200 μm, 100 μm, and 50 μm, respectively. (B) Energy-dispersive X-ray mapping of major elements present on the surface of bio-AgNPs CS/P407 hydrogel at × 250 magnification, scale bars = 100 μm.

The present study was to develop an *in situ*-forming CS/P407-based thermosensitive hydrogel loaded with bio-AgNPs using *E*. *camaldulensis* leaf extract. A promising drug-delivery hydrogel base was created with 0.5% chitosan and 24% poloxamer 407. The biosynthesized NPs were homogeneously mixed into a CS/P407-based hydrogel. The presence of bio-AgNPs in the hydrogel formulation was confirmed by UV-Vis spectroscopy (Fig 2A). The stability of the hydrogel formulation was evaluated at room temperature for 3 months, and the absorption peak (420 nm) of the NPs remained unchanged. The hydrogel formulation demonstrated rapid gelation under physiological conditions, as shown in Fig 2B. A recent study revealed that poloxamers-based hydrogel containing bio-AgNPs using *E*. *camaldulensis* extract exhibited a dense and robust three-dimensional structure and enhanced porosity within the hydrogel structure, compared with hydrogel alone [31]. Another study reported that chitosan-AgNPs hydrogel demonstrated embedded AgNPs stability and the production of porous inside three-dimensional hydrogel networks that facilitated gas exchange, promoting the healing process in a rat model of wound [32]. The structure morphology of the hydrogel formulation was examined using SEM, as shown in Fig 3A. The SEM micrographs illustrated a dense three-dimensional porous structure inside the hydrogel that is suitable for holding the amount of solution as well as transferring gases. EDX mapping images showed that bio-AgNPs were uniformly dispersed throughout the hydrogel framework (Fig 3B).

### Water content

**Fig 4:**
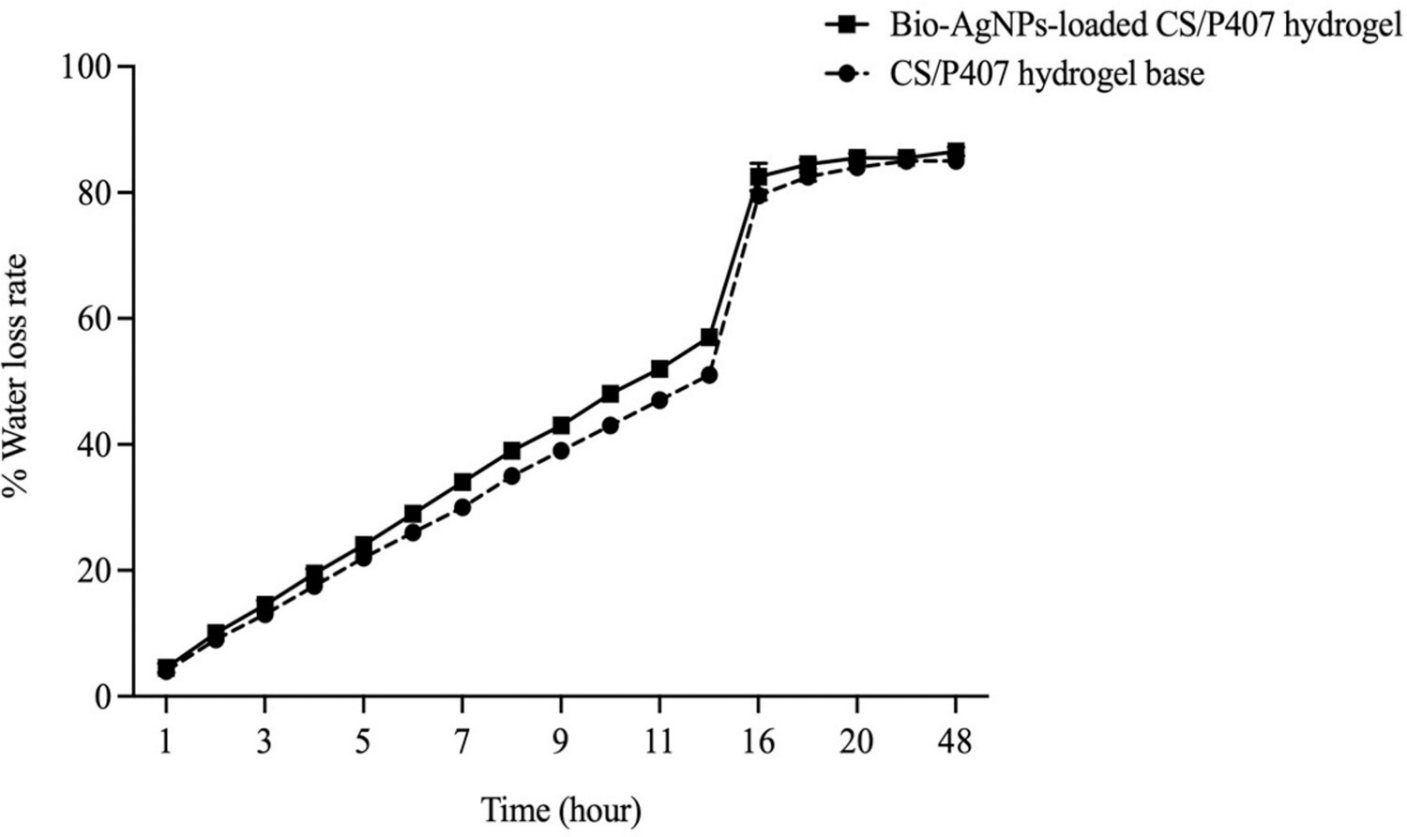
The water loss rate of chitosan/poloxamer 407-based thermosensitive hydrogel loaded with bio-AgNPs using *E*. *camaldulensis* leaf extract and CS/P407-based thermosensitive hydrogel alone at 37°C. Values represent three independent experiments and are expressed as mean ± SD.

Hydrogels’ ability to retain > 70% of water per weight is remarkable characteristic because they have a three-dimensional network of hydrophilic polymers. High-water-content hydrogels not only promote biocompatibility and mimic the hydration of tissues but also exhibit the strength of hydrogels due to the presence of cross-linking structures inside hydrogels, enhancing their potential to be used in biomedical applications [33, 34]. In this study, the water content of the hydrogels was determined by measuring the water loss rate of hydrogels at 37°C. The results demonstrated that both CS/P407-based hydrogel and bio-AgNPs-loaded CS/P407 hydrogel could retain water for up to 18 h (Fig 4). The water loss rates of the hydrogel base and the hydrogel formulation at 18 h were 83% and 85%, respectively, indicating that the hydrogels potentially reserve up to 85% of free water. A previous study demonstrated that CS/P407-based hydrogels could enhance sustained water retention after being maintained at 40°C for 12 h, improving wound hydration and decreasing pain. The capability of hydrogels to retain a large amount of water may be due to cross-linking between negatively charged polyoxyethylene networks of poloxamer networks and the primary amine groups of chitosan, resulting in the formation of three-dimensional porous networks [35].

### Antimicrobial susceptibility testing

The antimicrobial activity of bio-AgNPs and bio-AgNPs-loaded CS/P407 hydrogel was examined against clinical isolates of MDR pathogens and standard strains. The results revealed that bio-AgNPs and the hydrogel formulation demonstrated strong antimicrobial effects against Gram-positive and -negative bacteria and *C*. *albicans* (Table 1).

**Table 1:** Antimicrobial activity of nanocomposites used in this study.

Pathogen	MIC/MBC (μg/mL)		
	Bio-AgNPs	CS/P407 hydrogel	Bio-AgNPs + CS/P407 hydrogel
MDR *Staphylococcus aureus*	4/128	>256	4/32
MDR *Klebsiella pneumoniae*	8/32	>256	8/32
MDR *Acinetobacter baumannii*	8/16	>256	6/32
MDR *Pseudomonas aeruginosa*	8/16	>256	8/32
Methicillin-resistant *S*. *aureus*	4/64	>256	4/16
ATCC *Staphylococcus aureus*	4/32	>256	4/16
ATCC *Staphylococcus epidermidis*	4/16	>256	2/8
ATCC *Acinetobacter baumannii*	2/8	>256	4/8
ATCC *Pseudomonas aeruginosa*	2/16	>256	4/8
ATCC *Escherichia coli*	2/8	>256	4/16
	MIC/MFC (μg/mL)		
ATCC *Candida albicans*	8/64	>256	2/32

MIC, minimum inhibitory concentration; MBC, minimum bactericidal concentration; MFC minimum fungicidal concentration; MDR, multidrug-resistant; CS/P407, chitosan/poloxamer 407.

Minimum inhibitory concentration (MIC) and minimum bactericidal concentration (MBC) or minimum fungicidal concentration (MFC) values of bio-AgNPs were in a range from 2−8 μg/mL and 8−128 μg/mL, respectively. The hydrogel formulation showed activity against the pathogens with MIC and MBC or MFC values of 2−16 μg/mL and 8−32 μg/mL, respectively. In addition, CS/P407 hydrogel alone had no antibacterial activity when tested, even at the highest volume (256 μg/mL). It is observed that both the bio-AgNPs and the hydrogel formulation exhibited similar antibacterial activity, suggesting that the release of bio-AgNPs from the hydrogel networks was not disrupted or blocked by the CS/P407 hydrogel base. This study indicated that bio-AgNPs-loaded CS/P407 hydrogels are effective broad-spectrum antibacterial agents that could be related to their several mechanisms of action on the bacterial cell. For example, the green synthesis of AgNPs using *Piper retrofractum* fruit extract inhibited bacterial growth by causing bacterial cells to swell and shrink [36]. Further, bacterial membrane disruption and protein leakage were detected following treatment with bio-AgNP using aqueous *Rheum palmatum* root extract, resulting in a change in bacterial cell morphology and cell death [37].

### Cytotoxicity effects

**Fig 5:**
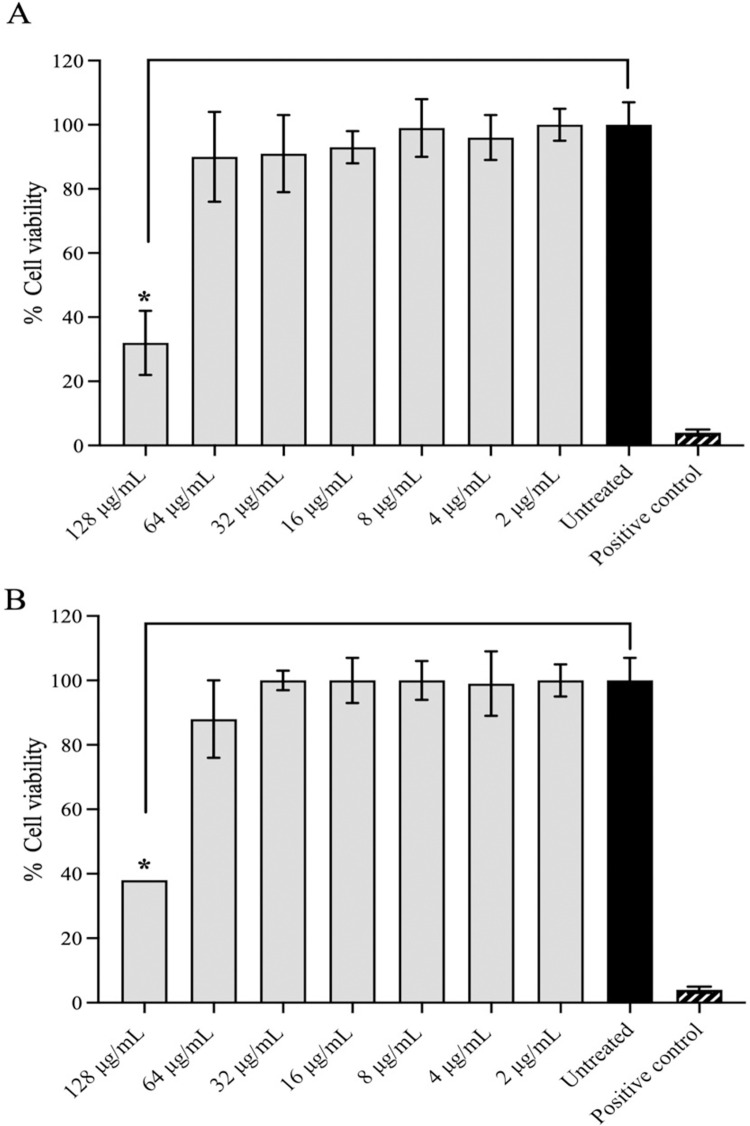
Cytotoxic effects of (A) bio-AgNPs using aqueous *E*. *camaldulensis* leaf extract and (B) CS/P407-based thermosensitive hydrogel loaded with bio-AgNPs on THP-1 macrophage cells. SDS (10%) was used as a positive control. Values represent three independent experiments and are expressed as mean ± SD. *Significant difference between treatment and control (p < 0.05).

Results showed that THP-1 cell viability was not significantly reduced by bio-AgNPs or the hydrogel formulation in the range between 2−64 μg/mL (Fig 5A and 5B). Following treatment, the percentage of viability of THP-1 cells ranged from 89−100%. On the other hand, a higher concentration (128 μg/mL) showed a significant decrease in viability by 50% (p > 0.05).

The presence of phytochemicals obtained from plant extracts that coat NPs may help to reduce toxic effects. A previous study demonstrated that green synthesis of AgNPs from *Rumex hymenosepalus* root extract had fewer toxicity effects on THP-1 macrophage cells than AgNO_3_ at the same concentrations [38]. Similarly, synthesized AgNPs using *Laurus nobilis* leaf extract did not exhibit remarkable cytotoxicity on THP-1 cells compared to conventional AgNPs. The toxicity impact difference was unrelated to a different cellular uptake rate [39].

### Immune response activity

**Fig 6:**
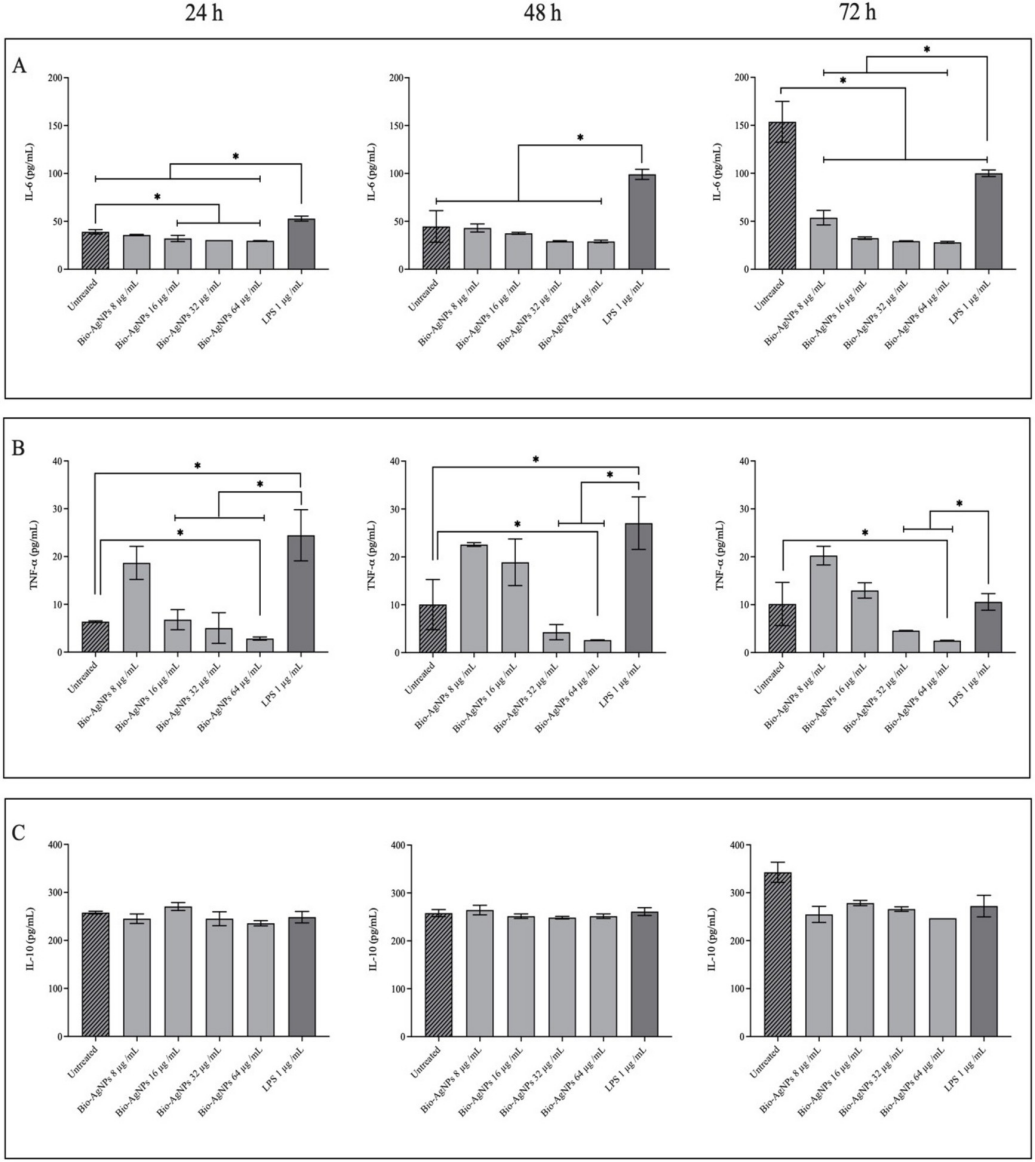
Inflammatory response of the bio-AgNPs used in this study. Cytokines were measured in the supernatants of THP-1 cells exposed to bio-AgNPs after 24, 48, and 72 h. (A) Interleukin-6, (B) Tumor necrosis factor-α, and (C) Interleukin-10. Values represent three independent experiments expressed as mean ± SD. LPS, lipopolysaccharide was used as a positive control. *Significant difference between treatments and the LPS-stimulated control group (p<0.05).

Confirming biomaterials’ safety profile in triggering cytokine release from immune cells is critical for applications. To examine the effects of bio-AgNPs and bio-AgNPs-loaded CS/P407 hydrogel on immune responses, the release of pro-inflammatory (IL-6 and TNF-α) and anti-inflammatory (IL-10) cytokines from THP-1 cells was investigated by ELISA assay. The cytokine production of THP-1 cells was assessed after 24, 48, and 72 h exposure to bio-AgNPs and the hydrogel formulation at concentrations varying between 8−64 μg/mL. The results demonstrated that bio-AgNPs at all concentrations did not significantly induce IL-6 throughout the testing compared to LPS-stimulated THP-1 cells (p > 0.05) (Fig 6A). Furthermore, most concentrations of bio-AgNPs significantly reduced IL-6 levels in THP-1 cells when compared to untreated cells after 24 and 72 h of exposure (p > 0.05). Following 24 h of treatment, bio-AgNPs at final concentrations varying between 16−64 μg/mL did not affect the increase of TNF-α; however, at 48 and 72 h, only the concentrations of 32 and 64 μg/mL could significantly reduce TNF-α levels when compared with the positive control (p > 0.05) (Fig 6B). While TNF-α production was markedly decreased after 24, 48, and 72 h of exposure to bio-AgNPs at 64 μg/mL, compared to untreated cells (p > 0.05) (Fig 6B). All bio-AgNPs concentrations could not upregulate the expression of the anti-inflammatory cytokine IL-10 (Fig 6C). A study from our lab revealed that lignin-capped AgNPs did not activate IL-6 or THF-α but could produce a significant amount of IL-10 in THP-1 cells after 24 h of exposure when compared with LPS-stimulated THP-1 cells [25]. Other research has confirmed that AgNPs synthesized by a plant extract stimulate pro-inflammatory cytokines, IL-6 and IL-8, at a lower level than conventional AgNPs on THP-1 cells after 24 and 48 h of treatment [39].

In conclusion, this study developed chitosan/poloxamer 407-based thermosensitive hydrogel loaded with bio-AgNPs using *Eucalyptus camaldulensis* leaf extract. Bio-AgNPs exhibited excellent colloid stability before and after incorporation into CS/P407-based hydrogel. The hydrogel formulation demonstrated rapid gelation under physiological conditions (33°C) with excellent water retention. Bio-AgNPs-loaded CS/P407 hydrogel showed antimicrobial activity against important human MRD strains, including Gram-positive and Gram-negative bacteria and *C*. *albicans*. In addition, bio-AgNPs-loaded CS/P407 hydrogel inhibited inducing pro-inflammatory cytokines but promoted anti-inflammatory cytokine production. The hydrogel formulation did not show toxic effects on THP-1 macrophage cells. The findings suggested that bio-AgNPs-loaded CS/P407 hydrogel with profound antimicrobial activity and anti-inflammatory properties could be an effective alternative biomaterial agent for chronically infected wound treatment.

## References

[1] Pasero D, Cossu AP, Terragni P. Multi-drug resistance bacterial infections in critically Ill patients admitted with COVID-19. Microorganisms. 2021;9: 1773. doi: 10.3390/microorganisms9081773 34442852 PMC8402127

[2] Mulani MS, Kamble EE, Kumkar SN, Tawre MS, Pardesi KR. Emerging strategies to combat ESKAPE pathogens in the era of antimicrobial resistance: A review. Front Microbiol. 2019;10: 539. doi: 10.3389/fmicb.2019.00539 30988669 PMC6452778

[3] Murray CJ, Ikuta KS, Sharara F, Swetschinski L, Robles Aguilar G, Gray A, et al. Global burden of bacterial antimicrobial resistance in 2019: a systematic analysis. The Lancet. 2022;399: 629–655. doi: 10.1016/S0140-6736(21)02724-0 35065702 PMC8841637

[4] Castanheira M, Messer SA, Rhomberg PR, Pfaller MA. Antifungal susceptibility patterns of a global collection of fungal isolates: results of the SENTRY Antifungal Surveillance Program (2013). Diagn Microbiol Infect Dis. 2016;85: 200–204. doi: 10.1016/j.diagmicrobio.2016.02.009 27061369

[5] Arendrup MC, Patterson TF. Multidrug-resistant Candida: Epidemiology, molecular mechanisms, and treatment. J Infect Dis. 2017;216: S445–S451. doi: 10.1093/infdis/jix131 28911043

[6] Khatoon Z, McTiernan CD, Suuronen EJ, Mah T-F, Alarcon EI. Bacterial biofilm formation on implantable devices and approaches to its treatment and prevention. Heliyon. 2018;4: e01067. doi: 10.1016/j.heliyon.2018.e01067 30619958 PMC6312881

[7] Snyder RJ, Bohn G, Hanft J, Harkless L, Kim P, Lavery L, et al. Wound biofilm: Current perspectives and strategies on biofilm disruption and treatment. Wounds. 2017;29: S1–S17. 28682297

[8] Rabin N, Zheng Y, Opoku-Temeng C, Du Y, Bonsu E, Sintim HO. Biofilm formation mechanisms and targets for developing antibiofilm agents. Future Med Chem. 2015;7: 493–512. doi: 10.4155/fmc.15.6 25875875

[9] Hengzhuang W, Wu H, Ciofu O, Song Z, Høiby N. In vivo pharmacokinetics/pharmacodynamics of colistin and imipenem in Pseudomonas aeruginosa biofilm infection. Antimicrob Agents Chemother. 2012;56: 2683–2690. doi: 10.1128/AAC.06486-11 22354300 PMC3346607

[10] Høiby N, Ciofu O, Johansen HK, Song Z, Moser C, Jensen PØ, et al. The clinical impact of bacterial biofilms. Int J Oral Sci. 2011;3: 55–65. doi: 10.4248/IJOS11026 21485309 PMC3469878

[11] Slavin YN, Asnis J, Häfeli UO, Bach H. Metal nanoparticles: understanding the mechanisms behind antibacterial activity. J Nanobiotechnology. 2017;15: 65. doi: 10.1186/s12951-017-0308-z 28974225 PMC5627441

[12] Martinez-Gutierrez F, Boegli L, Agostinho A, Sánchez EM, Bach H, Ruiz F, et al. Anti-biofilm activity of silver nanoparticles against different microorganisms. Biofouling. 2013;29: 651–660. doi: 10.1080/08927014.2013.794225 23731460

[13] Lakkim V, Reddy MC, Pallavali RR, Reddy KR, Reddy CV, Inamuddin, et al. Green Synthesis of Silver Nanoparticles and Evaluation of Their Antibacterial Activity against Multidrug-Resistant Bacteria and Wound Healing Efficacy Using a Murine Model. Antibiotics. 2020;9: 902. doi: 10.3390/antibiotics9120902 33322213 PMC7763323

[14] Li Y, Yang HY, Lee DS. Advances in biodegradable and injectable hydrogels for biomedical applications. J Control Release Off J Control Release Soc. 2021;330: 151–160. doi: 10.1016/j.jconrel.2020.12.008 33309972

[15] Pagano C, Giovagnoli S, Perioli L, Tiralti MC, Ricci M. Development and characterization of mucoadhesive-thermoresponsive gels for the treatment of oral mucosa diseases. Eur J Pharm Sci Off J Eur Fed Pharm Sci. 2020;142: 105125. doi: 10.1016/j.ejps.2019.105125 31682975

[16] Russo E, Villa C. Poloxamer hydrogels for biomedical applications. Pharmaceutics. 2019;11: 671. doi: 10.3390/pharmaceutics11120671 31835628 PMC6955690

[17] Abdeltawab H, Svirskis D, Sharma M. Formulation strategies to modulate drug release from poloxamer based in situ gelling systems. Expert Opin Drug Deliv. 2020;17: 495–509. doi: 10.1080/17425247.2020.1731469 32067500

[18] Fathalla Z, Mustafa WW, Abdelkader H, Moharram H, Sabry AM, Alany RG. Hybrid thermosensitive-mucoadhesive in situ forming gels for enhanced corneal wound healing effect of L-carnosine. Drug Deliv. 2022;29: 374–385. doi: 10.1080/10717544.2021.2023236 35068268 PMC8788381

[19] Rodrigues S, Dionísio M, López CR, Grenha A. Biocompatibility of chitosan carriers with application in drug delivery. J Funct Biomater. 2012;3: 615–641. doi: 10.3390/jfb3030615 24955636 PMC4030999

[20] Masood N, Ahmed R, Tariq M, Ahmed Z, Masoud MS, Ali I, et al. Silver nanoparticle impregnated chitosan-PEG hydrogel enhances wound healing in diabetes induced rabbits. Int J Pharm. 2019;559: 23–36. doi: 10.1016/j.ijpharm.2019.01.019 30668991

[21] Shah MZ, Guan ZH, Din AU, Ali A, Rehman AU, Jan K, et al. Synthesis of silver nanoparticles using *Plantago lanceolata* extract and assessing their antibacterial and antioxidant activities. Sci Rep. 2021; 11:20754. doi: 10.1038/s41598-021-00296-5 34675270 PMC8531362

[22] Hu D, Gao T, Kong X, Ma N, Fu J, Meng L, et al. Ginger (*Zingiber officinale*) extract mediated green synthesis of silver nanoparticles and evaluation of their antioxidant activity and potential catalytic reduction activities with Direct Blue 15 or Direct Orange 26. PLoS ONE, 2022;17:e0271408. doi: 10.1371/journal.pone.0271408 36006900 PMC9409512

[23] Wunnoo S, Paosen S, Lethongkam S, Sukkurd R, Waen‐ngoen T, Nuidate T, et al. Biologically rapid synthesized silver nanoparticles from aqueous *Eucalyptus camaldulensis* leaf extract: Effects on hyphal growth, hydrolytic enzymes, and biofilm formation in *Candida albicans*. Biotechnol Bioeng. 2021;118: 1578–1592. doi: 10.1002/bit.27675 33421102

[24] Nwabor OF, Singh S, Syukri DM, Voravuthikunchai SP. Bioactive fractions of Eucalyptus camaldulensis inhibit important foodborne pathogens, reduce listeriolysin O-induced haemolysis, and ameliorate hydrogen peroxide-induced oxidative stress on human embryonic colon cells. Food Chem. 2021;344: 128571. doi: 10.1016/j.foodchem.2020.128571 33221106

[25] Slavin YN, Ivanova K, Hoyo J, Perelshtein I, Owen G, Haegert A, et al. Novel Lignin-Capped Silver Nanoparticles against Multidrug-Resistant Bacteria. ACS Appl Mater Interfaces. 2021;13: 22098–22109. doi: 10.1021/acsami.0c16921 33945683

[26] CLSI C. Performance standards for antimicrobial susceptibility testing. Clin Lab Stand Inst. 2016;35: 16–38.

[27] Clayton KN, Salameh JW, Wereley ST, Kinzer-Ursem TL. Physical characterization of nanoparticle size and surface modification using particle scattering diffusometry. Biomicrofluidics. 2016;10: 054107. doi: 10.1063/1.4962992 27703593 PMC5035303

[28] Bhattacharjee S. DLS and zeta potential–What they are and what they are not? J Controlled Release. 2016;235: 337–351. doi: 10.1016/j.jconrel.2016.06.017 27297779

[29] Hamouda RA, Hussein MH, Abo-Elmagd RA, Bawazir SS. Synthesis and biological characterization of silver nanoparticles derived from the cyanobacterium Oscillatoria limnetica. Sci Rep. 2019;9: 13071. doi: 10.1038/s41598-019-49444-y 31506473 PMC6736842

[30] Singh P, Pandit S, Beshay M, Mokkapati VRSS, Garnaes J, Olsson ME, et al. Anti-biofilm effects of gold and silver nanoparticles synthesized by the Rhodiola rosea rhizome extracts. Artif Cells Nanomedicine Biotechnol. 2018;46: S886–S899. doi: 10.1080/21691401.2018.1518909 30422688

[31] Wunnoo S, Bilhman S, Waen‐ngoen T, Yawaraya S, Paosen S, Lethongkam S, et al. Thermosensitive hydrogel loaded with biosynthesized silver nanoparticles using Eucalyptus camaldulensis leaf extract as an alternative treatment for microbial biofilms and persistent cells in tissue infections. J Drug Deliv Sci Technol. 2022;74: 103588. doi: 10.1016/j.jddst.2022.103588

[32] Xie Y, Liao X, Zhang J, Yang F, Fan Z. Novel chitosan hydrogels reinforced by silver nanoparticles with ultrahigh mechanical and high antibacterial properties for accelerating wound healing. Int J Biol Macromol. 2018;119: 402–412. doi: 10.1016/j.ijbiomac.2018.07.060 30030078

[33] Means AK, Grunlan MA. Modern strategies to achieve tissue-mimetic, mechanically robust hydrogels. ACS Macro Lett. 2019;8: 705–713. doi: 10.1021/acsmacrolett.9b00276 33912358 PMC8077972

[34] Tran VT, Mredha MdTI, Jeon I. High-water-content hydrogels exhibiting superior stiffness, strength, and toughness. Extreme Mech Lett. 2020;37: 100691. doi: 10.1016/j.eml.2020.100691

[35] Lin S, Pei L, Zhang W, Shu G, Lin J, Li H, et al. Chitosan-poloxamer-based thermosensitive hydrogels containing zinc gluconate/recombinant human epidermal growth factor benefit for antibacterial and wound healing. Mater Sci Eng C. 2021;130: 112450. doi: 10.1016/j.msec.2021.112450 34702529

[36] Suci A, Akhmad S, Masruri M, Sutiman B. S. Characterization and antibacterial application of biosynthesized silver nanoparticles using Piper retrofractum Vahl fruit extract as bioreductor. J Appl Pharm Sci. 2022 [cited 9 Sep 2022]. doi: 10.7324/JAPS.2022.120311

[37] Arokiyaraj S, Vincent S, Saravanan M, Lee Y, Oh YK, Kim KH. Green synthesis of silver nanoparticles using *Rheum palmatum* root extract and their antibacterial activity against *Staphylococcus aureus* and *Pseudomonas aeruginosa*. Artif Cells Nanomedicine Biotechnol. 2017;45: 372–379. doi: 10.3109/21691401.2016.1160403 27023851

[38] Rodríguez-León E, Íñiguez-Palomares RA, Navarro RE, Rodríguez-Beas C, Larios-Rodríguez E, Alvarez-Cirerol FJ, et al. Silver nanoparticles synthesized with *Rumex hymenosepalus* extracts: effective broad-spectrum microbicidal agents and cytotoxicity study. Artif Cells Nanomedicine Biotechnol. 2018;46: 1194–1206. doi: 10.1080/21691401.2017.1366332 28826248

[39] Cascione M, Rizzello L, Manno D, Serra A, De Matteis V. Green Silver Nanoparticles Promote Inflammation Shutdown in Human Leukemic Monocytes. Materials. 2022;15: 775. doi: 10.3390/ma15030775 35160720 PMC8836503

