# Correction: Advanced biomaterial agent from chitosan/poloxamer 407-based thermosensitive hydrogen containing biosynthesized silver nanoparticles using *Eucalyptus camaldulensis* leaf extract

**DOI:** 10.1371/journal.pone.0315639

**Published:** 2024-12-09

**Authors:** Suttiwan Wunnoo, Ana C. Lorenzo-Leal, Supayang P. Voravuthikunchai, Horacio Bach

The word "hydrogel" is misspelled in the article title. The correct title is: Wunnoo S, Lorenzo-Leal AC, Voravuthikunchai SP, Bach H (2023) Advanced biomaterial agent from chitosan/poloxamer 407-based thermosensitive hydrogel containing biosynthesized silver nanoparticles using *Eucalyptus camaldulensis* leaf extract. PLOS ONE 18(10): e0291505. https://doi.org/10.1371/journal.pone.0291505.
